# Relief of Biofilm Hypoxia Using an Oxygen Nanocarrier: A New Paradigm for Enhanced Antibiotic Therapy

**DOI:** 10.1002/advs.202000398

**Published:** 2020-05-14

**Authors:** Dengfeng Hu, Lingyun Zou, Weijiang Yu, Fan Jia, Haijie Han, Ke Yao, Qiao Jin, Jian Ji

**Affiliations:** ^1^ MOE Key Laboratory of Macromolecule Synthesis and Functionalization of Ministry of Education, Department of Polymer Science and Engineering Zhejiang University Hangzhou Zhejiang Province 310027 P. R. China; ^2^ Eye Center Second Affiliated Hospital, School of Medicine Zhejiang University Hangzhou Zhejiang Province 310027 P. R. China

**Keywords:** antibiotic resistance, antibiotics, biofilms, hypoxia, perfluorohexane

## Abstract

Biofilms are chief culprits of most intractable infections and pose great threats to human health. Conventional antibiotic therapies are hypodynamic to biofilms due to their strong drug resistance, closely related with biofilm hypoxia. A new strategy for enhanced antibiotic therapy by relieving biofilm hypoxia is reported here. A two‐step sequential delivery strategy is fabricated using perfluorohexane (PFH)‐loaded liposomes (lip) as oxygen (O_2_) carriers (denoted as lip@PFH@O_2_) and commercial antibiotics. The results indicate that the two‐step sequential treatment exhibits much lower minimum bactericidal concentrations than the antibiotic treatment alone. In this design, the lip@PFH@O_2_ holds positively charged surface for better biofilm penetration. After penetrating into biofilm, oxygen can be released from lip@PFH@O_2_ by inches, which greatly relieves biofilm hypoxia. With the relief of hypoxia, the quorum sensing and the drug efflux pumps of bacteria are suppressed by restraining related gene expression, leading to the reduced antibiotic resistance. Furthermore, the in vivo experimental results also demonstrate that lip@PFH@O_2_ can effectively relieve biofilm hypoxia and enhance therapeutic efficacy of antibiotics. As a proof‐of‐concept, this research provides an innovative strategy for enhanced antibiotic therapy by relieving hypoxia, which may hold a bright future in combating biofilm‐associated infections.

## Graphical abstract

A perfluorohexane (PFH)‐loaded liposome nanocarrier (lip@PFH@O_2_) is developed for effective delivery of oxygen into biofilm. Such delivery of oxygen proved crucial in reducing biofilm hypoxia. The relief of biofilm hypoxia can ultimately decrease the drug resistance of biofilm to antibiotics through restraining the quorum sensing and drug efflux pumps activity by downregulating the related genes.

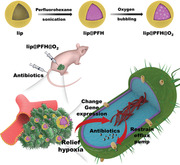

## 1 Introduction

Over 70% bacterial infections of human are caused by biofilms, such as intractable infections and chronic infections.^[^ ^1^, ^2^, ^3^ ^]^ In the past century, antibiotic therapy is the frequently used way to fight against bacterial infection.^[^ ^4^, ^5^ ^]^ Unfortunately, antibiotics exhibit significantly reduced bactericidal ability in combating bacterial biofilm infections due to the great drug resistance of biofilm.^[^ ^3^, ^6^, ^7^, ^8^ ^]^ Biofilms are well‐organized bacterial communities with self‐produced extracellular polymeric substances (EPS) consisting of polysaccharide, proteins, glycoprotein, and nucleic acids.^[^ ^8^, ^9^ ^]^ The protective EPS of biofilms can serve as effective physical and metabolic barriers, leading to high antibiotic resistance by restricting antibiotic penetration, inducing antibiotic inactivation via enzyme and chelation reaction, enhancing the quorum sensing, improving the activity of drug efflux pumps of bacteria, and so on. The biofilm‐encased bacteria can become up to 10–1000 times more resistant to antibiotics than planktonic bacteria.^[^ ^10^, ^11^ ^]^ In order to effectively eradicate biofilm‐encased bacteria, it is a conventional process to largely increase the dosage of antibiotics in clinic.^[^ ^5^, ^6^, ^12^ ^]^ However, higher dosage of antibiotics can not only result in severe side effect, such as liver and kidney damage, but also tremendously expedite the development of drug resistance. Thus, it is of great urgency to develop new strategies to improve the therapeutic performance of antibiotics for combating biofilm‐associated infections.

The EPS of biofilms leads to the establishment of complex organisms with stable gradients (oxygen, nutrient, pH, etc.), which comprises specific biofilm microenvironment.^[^ ^11^ ^]^ The hypoxia is a typical characteristic of biofilm, which results from the disruptive balance between the external oxygen supply and internal oxygen consumption by bacteria in biofilms.^[^ ^11^, ^13^, ^14^ ^]^ It is reported that biofilm hypoxia is an extremely important reason for the resistance of biofilm‐encased bacteria to many antibiotics.^[^ ^14^ ^]^ *P. aeruginosa* biofilm, a cause of many diseases, such as pulmonary cystic fibrosis, is a typical representative. It exhibits strong resistance to many antibiotics, especially penicillin and cephalosporin, which is closely related to the hypoxia. Therefore, the elimination of hypoxia might be an effective method to overcome antibiotic resistance in the biofilm treatment. However, how to relieve biofilm hypoxia by oxygen delivery or generation has never been reported and may represent an interesting future direction for enhanced antibiotic therapy in treating biofilm‐associated infections.

Perfluorohexane (PFH), which shows excellent biocompatibility and oxygen loading capacity, can act as an oxygen carrier,^[^ ^15^ ^]^ making it to be utilized as a blood substitute in clinic. In this work, we propose a sequential therapeutic strategy combining PFH and oxygen‐loaded liposome (denoted as lip@PFH@O_2_) with commercial antibiotics (aztreonam, ceftazidime, and piperacillin‐tazobactam) for combating biofilm‐associated infections. First, lip@PFH@O_2_ is designed for oxygen delivery to relieve biofilm hypoxia for reducing or overcoming antibiotic resistance. Then, antibiotics are administrated for eradicating biofilm‐encased bacteria. With weak positively charged surface and high oxygen‐carrying capacity, the lip@PFH@O_2_ is able to effectively penetrate into the biofilm and release oxygen, thereby resulting in remarkably elevated entire biofilm oxygenation. With the relief of biofilm hypoxia, the minimum bactericidal concentration (MBC) of antibiotics reduces several folds, suggesting the enhanced antibacterial effect of antibiotics with the help of hypoxic relief. The gene sequencing indicates the relief of biofilm hypoxia could restrain the quorum sensing and the activities of drug efflux pumps by downregulating the related genes expression, which helps to remarkably decrease the biofilm drug resistance and improve the therapeutic efficacy of antibiotics. As far as we know, it is the first example that biofilm hypoxia can be relieved by an oxygen delivery system for enhanced antibiotic therapy, which provides a new avenue to combat biofilm‐associated infections.

## 2 Results and Discussion

### 2.1 Synthesis and Characterization of lip@PFH@O_2_

**Figure 1:**
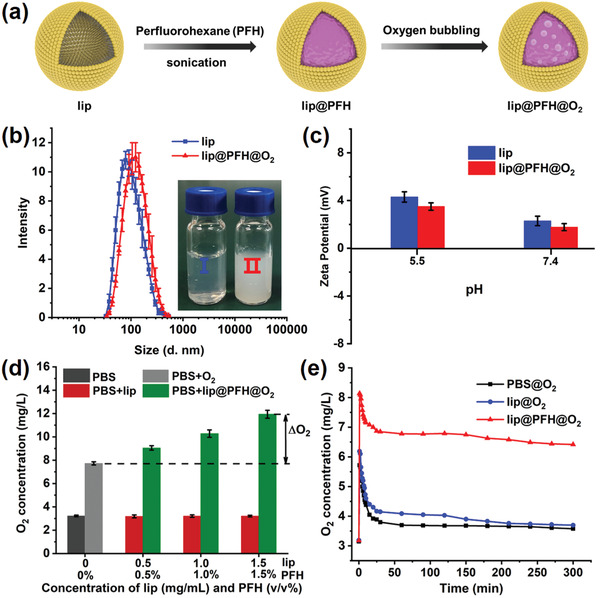
a) Schematic diagram of preparation process of lip@PFH@O_2_; b) Hydrodynamic diameter of lip and lip@PFH@O_2_. Inset: photos of lip and lip@PFH@O_2_ solution; c) Zeta potentials of lip and lip@PFH@O_2_ at different pH values; d) The measurement of oxygen loading capacity of lip@PFH@O_2_. Oxygen concentration in 5 mL phosphate buffered saline (PBS, 10 × 10^−3^ m) before and after adding 5 mL of lip@PFH@O_2_ with different concentrations. ΔO_2_: Enhanced O_2_ concentration; e) The oxygen concentration changes of PBS solution after adding PBS@O_2_ solution, lip@O_2_ solution, and lip@PFH@O_2_ solution, respectively.

lip@PFH@O_2_ with high oxygen loading capacity is prepared by membrane‐sonic method and subsequent oxygen bubbling (**Figure** **1**). The successful preparation of PFH‐loaded liposomes (denoted as lip@PFH) is confirmed by gas chromatography‐mass spectrometry due to the appearance of same mass peak and efflux time with PFH (Figure S1, Supporting Information). Then, lip@PFH@O_2_ is obtained by treating lip@PFH with oxygen bubbling. The lip@PFH@O_2_ exhibits spherical structure with hydrodynamic diameter of 120 ± 3.5 nm (Figure 1 and Figure S2a, Supporting Information). Due to the existence of the cationic phospholipid molecules, the lip@PFH@O_2_ possesses positively charged surface (zeta potential, 3.51 ± 0.31 mV) at acidic biofilm pH (Figure 1), which is beneficial to promote the penetration of nanoparticles into biofilms. The liposomes are widely used nanocarriers for both hydrophobic and hydrophilic drugs. As a kind of hydrophobic substance, PFH can be loaded into the liposomes easily and the excellent stability of lip@PFH and lip@PFH@O_2_ has been demonstrated in previous research.^[^ ^16^ ^]^

### 2.2 Oxygen Loading and Releasing Ability of Lip@PFH@O_2_

PFH has excellent oxygen‐dissolving ability owing to the strong van der Waals interaction between PFH and oxygen. Thus, lip@PFH@O_2_ may serve as an excellent oxygen carrier to deliver and release oxygen in the oxygen deficient biofilm. First, the oxygen loading capacity of lip@PFH@O_2_ is measured to be 2.81 ± 0.13 mg g^−1^ lip or 1.0% (v/v) PFH in the nanoparticles (Figure 1). Subsequently, the oxygen release behavior is further investigated. The phosphate buffered saline (PBS) solution, blank lip solution, and lip@PFH solution saturated with oxygen are added into the deoxygenated PBS. The oxygen concentrations of these solutions are measured by oxygen meter in real time. Different from the lip@O_2_ solution showing sharp oxygen decreasing in the first 30 min, the lip@PFH@O_2_ solution exhibits much slower oxygen decreasing and a higher final oxygen concentration even after 300 min (Figure 1). Hence, the above results demonstrate the lip@PFH has excellent oxygen loading ability and oxygen can be readily released in the hypoxic environment.

### 2.3 Biofilm Penetration Ability of lip@PFH@O_2_

It is reported that positively charged nanoparticles can efficiently penetrate into biofilms and adhere to the bacteria owing to the negatively charged bacterial membrane.^[^ ^2^ ^]^ Consequently, we investigate the penetration ability of lip@PFH by loading chlorin e6 (Ce6) fluorescent dye into the lip@PFH for confocal laser scanning microscopy (CLSM) observation. After adding the lip@PFH@Ce6 into *P. aeruginosa* biofilm for 1 h, the red fluorescence of Ce6 in biofilm can be observed clearly even in the bottom of the biofilm (Figure S3, Supporting Information), indicating the excellent penetration ability of lip@PFH@Ce6 owing to the positively charged surface of nanoparticles.

### 2.4 In Vitro Biofilm Hypoxia Relieved by lip@PFH@O_2_

**Figure 2:**
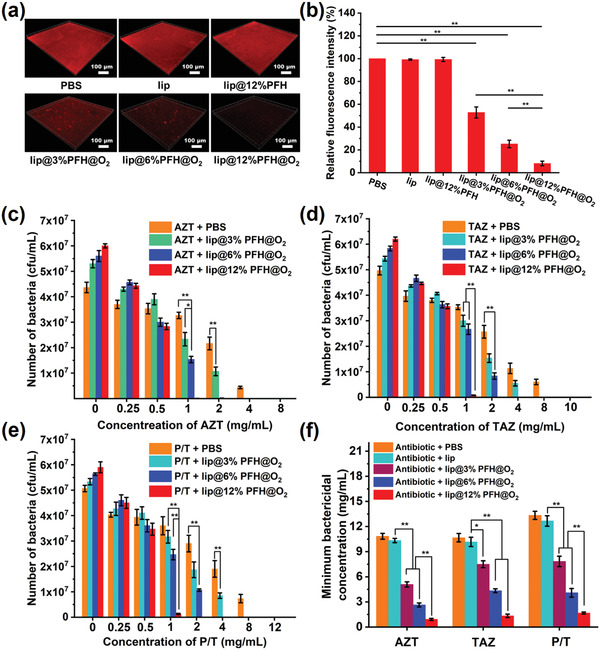
a) The representative immunofluorescence images of *P. aeruginosa* biofilm with different treatments for 2 h and then stained by hypoxyprobe; b) Quantification of fluorescence intensity in different groups shown in (a); The results of standard plate counting assay of c) *P. aeruginosa* biofilm treated with aztreonam (AZT, a representative antibiotic of penicillin) and lip@PFH@O_2_ with different oxygen concentrations, d) *P. aeruginosa* biofilm treated with ceftazidime (TAZ, a representative antibiotic of cephalosporin) and lip@PFH@O_2_ with different oxygen concentrations, and e) *P. aeruginosa* biofilm treated with piperacillin‐tazobactam (P/T, a kind of representative mixed antibiotics) and lip@PFH@O_2_ with different oxygen concentrations; f) Minimum bactericidal concentration of three kinds of antibiotics (aztreonam, AZT; ceftazidime, TAZ; piperacillin‐tazobactam, P/T) for *P. aeruginosa* biofilm.

Given the high oxygen loading capacity and the excellent biofilm penetration ability, the lip@PFH@O_2_ is hypothesized to be able to deliver and release oxygen in the biofilms, thus relieving the hypoxia of biofilm. The biofilm oxygenation enhancement effects after different treatments are investigated by a hypoxyprobe (pimonidazole hydrochloride) for immunofluorescence staining. The hypoxic signal of biofilm treated with lip or lip@PFH is almost unchanged compared to PBS control. However, significantly reduced hypoxic signal is detected upon the *P. aeruginosa* biofilm is treated with lip@PFH@O_2_, indicating effectively relieved hypoxic microenvironment. More interestingly, compared with lip@3%PFH@O_2_ (3%PFH, v/v%) and lip@6%PFH@O_2_ (6%PFH, v/v%) nanoparticles, the lip@12%PFH@O_2_ (12%PFH, v/v%) nanoparticles show much better oxygenation enhancement effect for *P. aeruginosa* biofilm (**Figure** **2**b), due to the much higher loading content of PFH and oxygen.

### 2.5 In Vitro Bactericidal Effect of Antibiotics

After biofilm hypoxia is relieved by lip@PFH@O_2_, it is very interesting to know whether the bactericidal effect of antibiotics can be enhanced in biofilm treatment. The bactericidal ability of aztreonam (AZT, a representative antibiotic of penicillins), ceftazidime (TAZ, a representative antibiotic of cephalosporins), and piperacillin‐tazobactam (P/T, a kind of representative mixed antibiotics) against *P. aeruginosa* biofilm with or without pretreatment of lip@PFH@O_2_ is further investigated by standard plate counting assay. Compared with the treatment of antibiotics only, the sequential treatment with lip@PFH@O_2_ and antibiotics shows better bactericidal effect at the same antibiotic concentration. The antibiotics combining with lip@12%PFH@O_2_ exhibit much stronger bactericidal capability than the antibiotics with lip@6%PFH@O_2_ and the antibiotics with lip@3%PFH@O_2_ even at same antibiotic concentration (Figure 2d,e). Therefore, the hypoxia level of the biofilm can significantly influence the therapeutic efficacy of antibiotics. In addition, as shown in Figure 2, when the combination therapy of lip@PFH@O_2_ and antibiotics is used for biofilm treatment, the minimum bactericidal concentrations (MBC, at which the bactericidal rate came up to at least 99.9%) of the above three antibiotics are largely reduced. For instance, the MBC of AZT with lip@3%PFH@O_2_ was 5.08 ± 0.31 mg mL^−1^, which is only 54% of the MBC of AZT only (10.83 ± 0.35 mg mL^−1^). More interestingly, the MBC of antibiotics exhibits obvious negative correlation with oxygen amount. The MBC of AZT (0.92 ± 0.12 mg mL^−1^) combining with lip@12%PFH@O_2_ is merely 8.5% of that treated with AZT only (10.83 ± 0.35 mg mL^−1^). Moreover, the MBC of TAZ (1.33 ± 0.20 mg mL^−1^) combining with lip@12%PFH@O_2_ or P/T (1.67 ± 0.12 mg mL^−1^) combining with lip@12%PFH@O_2_ are also less than 13% of that treated with TAZ (10.67 ± 0.50 mg mL^−1^) only or P/T (13.33 ± 0.49 mg mL^−1^) only. All these results demonstrate that relieving biofilm hypoxia can effectively enhance the bactericidal ability of antibiotics, probably due to the decrease of drug resistance of bacteria in biofilm. The 3‐(4,5‐dimethyl‐thiazol‐2‐yl)‐2,5‐diphenyl tetrazolium bromide (MTT) assay indicates the negligible cytotoxicity of these antibiotics to healthy tissues at the concentrations in our following researches (Figure S4, Supporting Information).

### 2.6 Biofilm Hypoxia Relieved by lip@PFH@O_2_ and Bactericidal Effect of Antibiotics in Subcutaneous *P. aeruginosa* Infection Model

**Figure 3:**
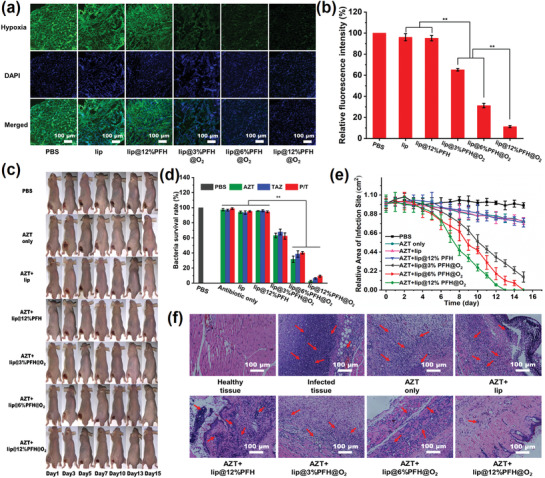
a) Representative immunofluorescence images of *P. aeruginosa* biofilm stained by the hypoxyprobe. The nuclei and hypoxia area are stained with DAPI (blue) and anti‐pimonidazole antibody (green), respectively; b) Quantification of biofilm hypoxia for different groups shown in immunofluorescence images of biofilm in (a); c) The representative photographs of *P. aeruginosa* (10^8^ cfu mL^−1^) infected mice within 15 d after different treatments; d) Related quantitative results of standard plate counting assay after different treatments with same antibiotic concentration (1 mg mL^−1^) in the subcutaneous *P. aeruginosa* biofilm infection model; e) The area change of *P. aeruginosa* biofilm infection sites of the mice after different treatments in the subcutaneous *P. aeruginosa* biofilm infection model; f) Histological photographs of infected skin tissues after different treatments, the inflammatory cells marked by red arrows (on 15th day). Scale bar: 100 µm.

Encouraged by the outstanding bactericidal effect of the sequential therapy combining with lip@PFH@O_2_ and antibiotics in vitro, the bactericidal ability of such sequential therapy strategy is evaluated by subcutaneous *P. aeruginosa* biofilm infection model on nude mice. After in situ injection of lip@PFH@O_2_ for 2 h, the *P. aeruginosa* biofilm infected site is cut down and then frozen for immunofluorescence staining assay to evaluate their changes of hypoxia. As shown in **Figure** **3b**, different from the strong green fluorescence in the groups treated with PBS, lip, and lip@PFH, only very weak green fluorescence can be observed in the group treated with lip@PFH@O_2_, indicating lip@PFH@O_2_ plays an important role in relieving the hypoxia in the biofilm infected sites. Furthermore, the decrease of hypoxic level in biofilm infected sites is positively correlated to the oxygen loading amount of lip@PFH@O_2_, which is in accordance with the in vitro results.

Next, the bactericidal ability of antibiotics combining with lip@PFH@O_2_ is investigated in subcutaneous *P. aeruginosa* biofilm infection model on nude mice. Compared with bacterial survival rates in abscess treated with antibiotics (1 mg mL^−1^, 100 µL) only, the bacterial survival rates in abscess with sequential treatment of lip@PFH@O_2_ (100 µL) and antibiotics (1 mg mL^−1^, 100 µL) is much lower (Figure 3). For instance, the bacterial survival rate in abscess treated with AZT and lip@12%PFH@O_2_ (2.45 ± 1.07%) is only 140th of that treated with AZT only (97.32 ± 1.54%). Moreover, the bacterial survival rate in the group of TAZ and lip@12%PFH@O_2_ (6.28 ± 0.89%) and the bacterial survival rate in the group of P/T and lip@12%PFH@O_2_ (9.25 ± 1.25%) are also less than one‐tenth of that treated with TAZ only (96.78 ± 1.25%) or P/T only (98.77 ± 1.1%), respectively. These results indicate the antibiotics exhibit enhanced therapeutic effect with the help of lip@PFH@O_2_. Different from the abscess treated with AZT only remaining red and inflamed (Figure 3), the abscess in the mice disappears on the 15th day after treatment with AZT and lip@12%PFH@O_2_ (Figure 3). The body weight of mice treated with PBS, AZT only, AZT with lip nanoparticles, and AZT with lip@12%PFH decreases with time going by, while the body weight of mice remains unchanged after the sequential treatment of lip@PFH@O_2_ and antibiotics (Figure S5, Supporting Information), suggesting the excellent therapeutic effect. In addition, the inflammation cells are greatly reduced (Figure 3) on the 15th day after treatment with AZT and lip@12%PFH@O_2_. Whereas the abscess treated with AZT only is still severe at this moment, which demonstrates that the lip@PFH@O_2_ can significantly improve the bactericidal effects of antibiotics.

### 2.7 Biofilm Hypoxia Relieved by lip@PFH@O_2_ and Bactericidal Effect of Antibiotics in Chronic Pneumonia Model

**Figure 4:**
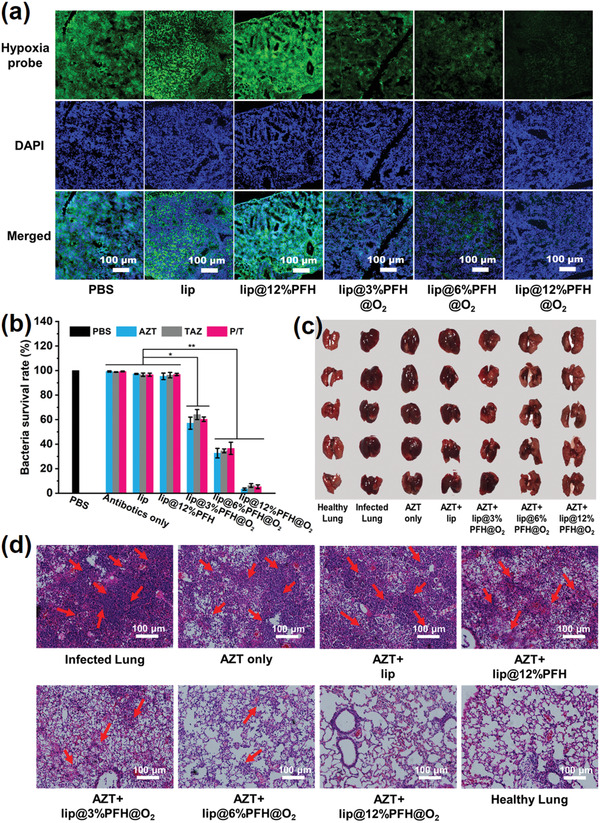
a) Representative immunofluorescence images of lungs infected by *P. aeruginosa* biofilm with different treatments stained by the hypoxyprobe. The nuclei and hypoxia areas are stained with DAPI (blue) and anti‐pimonidazole antibody (green), respectively. Scale bar: 100 µm; b) Related quantitative results of standard plate counting assay after different treatments with same antibiotic concentration (1 mg mL^−1^) in the *P. aeruginosa* biofilm infected chronic pneumonia model; c) Digital photographs of *P. aeruginosa* biofilm infected lungs treated with different nanoparticles with same aztreonam concentration (AZT, 1 mg mL^−1^) on 2nd day; d) Histological photographs of infected lungs treated with different nanoparticles with same aztreonam concentration (AZT, 1 mg mL^−1^) on 2nd day, the inflammatory cells marked by red arrows.

The in vivo antibacterial behavior of antibiotics with lip@PFH@O_2_ is further evaluated by the *P. aeruginosa* biofilm infected chronic pneumonia model. The *P. aeruginosa* suspension (3 × 10^6^ cfu mL^−1^, 100 µL) is infused to the lung of ICR mice by injector with soft needle. After 24 h, the lung is infected by *P. aeruginosa* biofilm and the chronic pneumonia is formed. Next, the lip@PFH@O_2_ solution (100 µL) is infused to the lung by injector with soft needle. 2 h later, the lung is cut out and injected with hypoxyprobe solution. Then, the change of hypoxia in *P. aeruginosa* infected lungs is investigated by the immunofluorescence staining assay. As shown in **Figure** **4**, with the increase of oxygen amount loaded in lip@PFH@O_2_, the green fluorescence gradually weakens, suggesting the hypoxia of lungs infected by biofilm is greatly alleviated. After sequentially infusing lip@PFH@O_2_ and AZT via respiratory tract to lungs for 24 h, the bactericidal effect is evaluated by the standard plate counting assay. No distinct antibacterial effect can be observed if mice are treated with AZT alone, AZT and lip, or AZT and lip@12%PFH. In contrast, the bacterial survival rates in lungs under the treatment of AZT and lip@PFH@O_2_ are obviously lower than that treated with PBS control (Figure 4). Moreover, there is no significant difference in appearance between the healthy lungs and the lungs treated with AZT and lip@12%PFH@O_2_ after 2 d (Figure 4), indicating the excellent bactericidal ability of the sequential therapy using AZT and lip@12%PFH@O_2_. Moreover, if TAZ or P/T is used in the sequential therapy with lip@PFH@O_2_, remarkably enhanced bactericidal effect is also observed in the treatment of lung infections (Figure 4), which also confirms the necessity of the pretreatment of lip@PFH@O_2_. At the same time, the lungs are stained by the hematoxylin and eosin (H&E) to further evaluate the inflammation after different treatments. As shown in Figure 4, the inflammation cells in the lungs treated with AZT with lip@12%PFH@O_2_ dramatically reduce after 2 d treatment, further demonstrating that relieving biofilm hypoxia can significantly improve the therapeutic efficacy of antibiotics in the treatment of lung infections.

### 2.8 The Mechanism of the Reduced Antibiotic Resistance of Biofilm with the Treatment of lip@PFH@O_2_

**Figure 5:**
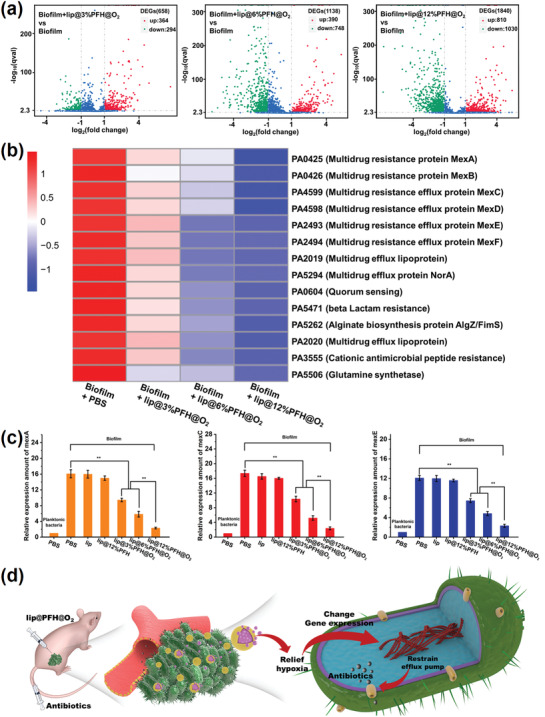
a) Volcano plot of gene expression differences between *P. aeruginosa* biofilm and *P. aeruginosa* biofilm treated with lip@3%PFH@O_2_, lip@6%PFH@O_2_, or lip@12%PFH@O_2_, respectively; b) Heat cluster plot of gene expression differences among *P. aeruginosa* biofilm treated with PBS, lip@3%PFH@O_2_, lip@6%PFH@O_2_, and lip@12%PFH@O_2_; c) The qRT‐PCR gene detection of relative expression amount of mexA, mexC, and mexE in *P. aeruginosa* bacteria in biofilm treated with different groups in vitro; d) The schematic diagram of treatment process of biofilm infection by using the antibiotics combining with lip@PFH@O_2_.

In order to investigate the mechanism of the reduced antibiotic resistance of biofilm with the treatment of lip@PFH@O_2_, the gene expression of *P. aeruginosa* in biofilm with different treatments is investigated by gene sequencing. It is obvious that the gene expression of *P. aeruginosa* in biofilm exhibits huge difference after treating with lip@PFH@O_2_. As can be seen in the volcano plot (**Figure** **5**), 658 genes of bacteria in biofilm are changed after the treatment of lip@3%PFH@O_2_ and 1138 genes of bacteria in biofilm are changed after treatment of lip@6%PFH@O_2_, whereas, about 1840 genes of bacteria in biofilm are changed after the biofilm treated with lip@12%PFH@O_2_. This suggests that reducing the biofilm hypoxia can effectively change the gene expression of *P. aeruginosa* in biofilm. More importantly, with the alleviation of biofilm hypoxia, the expression of genes related to multidrug resistance and quorum sensing is largely downregulated, which implies that drug resistance is greatly decreased by the relief of biofilm hypoxia (Figure 5). Moreover, the expression of three major genes closely related with multidrug resistance efflux pumps (mexA, mexC, and mexE) is further detected by the quantitative reverse transcription polymerase chain reaction (qRT‐PCR) measurements. Compared with the planktonic *P. aeruginosa*, the *P. aeruginosa* in hypoxic biofilms shows much higher gene expression of mexA, mexC, and mexE (Figure 5 and Figure S6, Supporting Information), indicating strong drug resistance of *P. aeruginosa* biofilms. After treatment with lip@PFH@O_2_, the gene expression of mexA, mexC, and mexE descend sharply. For example, the relative expression amount of mexA in the biofilm treated with lip@12%PFH@O_2_ is only one‐seventh of that treated with PBS. These results indicated that the lip@PFH@O_2_ can effectively relieve the biofilm hypoxia, leading to the great decrease of drug resistance by downregulating the expression of multidrug resistance genes. In addition, after treatment with lip@PFH@O_2_, the downregulation of expression of multidrug resistance genes (mexA, mexC, and mexE) is also observed in the subcutaneous *P. aeruginosa* biofilm infection model (Figure S7, Supporting Information). The above results indicate that relieving hypoxia can restrain the quorum sensing and drug efflux pumps activity by downregulating the related genes, ultimately decreasing the drug resistance of biofilm to antibiotics (Figure 5).

Hypoxia is a typical characteristic of almost all biofilms and is an important reason for antibiotic resistance of biofilm‐encased bacteria. The relief of biofilm hypoxia can be considered as a general strategy for enhanced antibiotic therapy in treating many kinds of biofilms. All strategies that can deliver or generate oxygen to relieve biofilm hypoxia can be used to reduce antibiotic resistance in biofilm treatment.

## 3 Conclusion

In summary, a new anti‐biofilm strategy is developed by relieving biofilm hypoxia, which can remarkably enhance the bactericidal ability of antibiotics by reducing drug resistance in treating biofilm‐associated infections. The two‐step therapeutic strategy is adopted by firstly relieving biofilm hypoxia using lip@PFH@O_2_ and subsequent antibiotic therapy to eradicate biofilm‐encased bacteria. The lip@PFH@O_2_ with positively charged surface exhibits excellent biofilm penetration ability based on the electrostatic interaction with negatively charged bacteria in biofilm. It is noteworthy that the lip@PFH@O_2_ shows continuous oxygen‐releasing behavior in hypoxic environment. The released oxygen can relieve the biofilm hypoxia, which can effectively reduce antibiotic resistance. Therefore, to achieve the same bactericidal effect, the concentration of antibiotics combining with lip@PFH@O_2_ is much lower than that of antibiotics alone, which has not only been testified by the in vitro biofilms, but also been verified by the in vivo biofilm infected subcutaneous abscess model and biofilm‐associated lung infection model. The possible mechanism of reduced antibiotic resistance of biofilm is that relieving biofilm hypoxia can restrain the quorum sensing and drug efflux pumps activity via downregulating the related genes. The strategy of decreasing biofilm drug resistance by relieving hypoxia may hold bright future in combating biofilm‐associated infections.

## 4 Experimental Section

##### Synthesis of lip@PFH@O_2_

First, DOTAP:DOPE:HSPC:DSPE‐PEG2000:Chol (a total of 30 mg) with a 10:20:40:10:20 molar ratio were dissolved in 10 mL chloroform. The solvent was then removed by rotary evaporation at 37 °C to form lipid films. Next, 10 mL deionized water was added into the flask and then they were put into the water bath ultrasonic for 10 min sonication to peel off the film. Then, 300 µL PFH was added into the solution following a 15 min sonication by ultrasonic probe in an ice bath. After the sonication, lip@3%PFH was obtained. At last, the lip@3%PFH‐O_2_ can be harvest by bubbling oxygen gas into lip@3%PFH solution for 5 min. In addition, the lip@PFH@O_2_ with different PFH amount (such as lip@6%PFH@O_2_ and lip@12%PFH@O_2_) can be obtained by changing the PFH materials input.

##### Measurement of Hydrodynamic Diameters and Zeta Potentials of lip@PFH@O_2_

Hydrodynamic diameters of lip@PFH@O_2_ were measured at 25 °C using a Delsa Nano C Particle Analyser (Beckman Coulter Ireland Inc.) in PB (10 × 10^−3^ m) with pH 5.5 or 7.4. Moreover, zeta potentials of lip@PFH@O_2_ were measured by using the Zetasizer.

##### Measurement of O_2_ Loading Capacity of lip@PFH

At first, the deoxygenated PBS (5 mL) was added into a three‐necked flask (25 mL) which is sealed by rubber plugs. The oxygen concentration of the solution was measured by the probe of portable dissolved oxygen meter (Rex, JPF‐605B, China), which is inserted through rubber plug into the flask. Subsequently, the oxygen‐saturated lip@PFH@O_2_ solutions (5 mL) with different concentrations were injected via syringe into the closed flask and then the oxygen concentration was recorded at the time of oxygen equilibrium.

##### Measurement of O_2_ Releasing Behavior of lip@PFH@O_2_

First, the deoxygenated phosphate buffer solution (5 mL) was added into the flask (25 mL) full of nitrogen. Next, the oxygen concentration was measured in real time by the oxygen electrode probe, which is inserted into the flask. The oxygen‐saturated lip@3%PFH@O_2_ solution (5 mL) was injected into the flask, and then the oxygen concentration was measured in the whole process. Moreover, the PBS solution with oxygen saturation (5 mL) and the solution of lip nanoparticles with oxygen saturation (5 mL) were also measured as controls.

##### In Vitro Immunofluorescence Staining of Biofilm by Hypoxyprobe

First, different nanoparticles (100 µL) were added into the *P. aeruginosa* biofilm cultured on glass slides for 2 h, respectively. Then, the nanoparticles were washed off by PBS. Subsequently, the pimonidazole hydrochloride (100 µL) was added into the *P. aeruginosa* biofilm for 90 min. Then, the primary antibodies which can specifically bind to the pimonidazole hydrochloride were added into the biofilm for 1 h. Subsequently, the second antibodies with fluorescent molecular gets into the biofilm to bind to the primary antibodies for 1 h. Next, the biofilm was washed by the PBS solution to clear the extra second antibodies which do not bind to the primary antibodies. Finally, all biofilm slices were observed under a fluorescence microscope (Olympus IX81).

##### In Vitro Bactericidal Effect of Antibiotics by Standard Plate Counting Assays

First, the lip@PFH@O_2_ (100 µL) with various PFH amounts were added into the biofilm cultured in the 24‐well plates for incubation for 2 h. Next, the antibiotics (AZT, TAZ, and P/T as representatives, 100 µL) with different concentrations were put into the biofilm for incubation for 8 h. Then, the biofilms were dispersed by the sonication. Subsequently, the suspensions were serially diluted with sterile PBS and 100 µL diluted samples were spread on the LB agar medium plates. The colonies formed were counted after incubation at 37 °C for 12 h. In addition, the minimum bactericidal concentration of different antibiotics was also obtained by the standard plate counting assays according to the above methods.

##### *P. aeruginosa* Biofilm Infected Subcutaneous Abscess Mouse Model

All animal experiments were performed according to the “Principles of Laboratory Animal Care” (NIH publication no. 86‐23, revised 1985) and the guidelines for Animal Care and Use Committee, Zhejiang University. Healthy male BALB/c nude mice (16–20 g each) which were obtained from animal center of Zhejiang Academy of Medical Sciences were employed to conduct the animal study. The subcutaneous abscess was created in each mouse by injecting *P. aeruginosa* (2 × 10^8^ cfu mL^−1^) with a dose of 100 µL in the left side of the mice to initiate infection. After 24 h, the *P. aeruginosa* biofilm infected subcutaneous abscess was formed.

##### In Vivo Bactericidal Effect of Nanoparticles in Subcutaneous Abscess Mouse Model

At first, the lip@PFH@O_2_ (100 µL) with various PFH amounts was in situ injected into the abscess. After 2 h, the antibiotics (100 µL, 1 mg mL^−1^) were injected into the abscess by caudal vein. After 24 h, the mice were sacrificed and the bacteria in the abscess were counted by the standard plate counting assays. Moreover, the infection area changes of each mouse were monitored and recorded regularly. After 15 d treatment, the mice were sacrificed and the infection sites were excised from the mice, and then they were fixed in 4% formaldehyde and embedded in paraffin. H&E staining assay was employed to further evaluate the treatment effect.

##### *P. aeruginosa* Biofilm Infected Chronic Pneumonia Mouse Model

The *P. aeruginosa* suspension (3 × 10^6^ cfu mL^−1^, 100 µL) was infused to the lung of ICR mice by injector with soft needle. After 24 h, the lung was infected by *P. aeruginosa* biofilm and the chronic pneumonia was formed.

##### In Vivo Immunofluorescence Staining of Biofilm Infected Pneumonia by Hypoxyprobe

Different nanoparticles (100 µL) were infused into the lung of mice by respiratory tract. After 2 h, the mice were sacrificed and the lungs were cut out and the pimonidazole hydrochloride (100 µL) was injected into the lungs for a 90 min incubation. After incubation, the lungs were frozen and sliced, and then the slices were stained. Finally, the slices were observed under a fluorescence microscope (Olympus IX81).

##### In Vivo Bactericidal Effect of Antibiotics in Biofilm Infected Pneumonia Mouse Model

First, the lip@PFH@O_2_ (100 µL) with various PFH amounts was infused to the lungs of mice. 2 h later, the antibiotics (100 µL, 1 mg mL^−1^) were infused into the lungs by respiratory tract. After 24 h, five mice in each group were sacrificed and the bacteria in lungs were counted by the standard plate counting assays. Moreover, after 2 d treatment, the lungs of another five mice in each group were obtained and then they were fixed in 4% formaldehyde and embedded in paraffin. H&E staining assay was employed to further evaluate the treatment effect.

##### Gene Transcriptome Test

At first, different nanoparticles (100 µL) were added into the *P. aeruginosa* biofilm cultured in 24‐well plates for 2 h, respectively. The biofilm suspension was taken out by pipette after a 10 min ultrasound, and then the bacteria in biofilm were obtained by centrifugation (3000 rpm, 5 min). Subsequently, the obtained bacteria were sent to Novogene Co., Ltd. (Beijing, China) to measure the gene expression changes. Before sending the samples, the bacteria amount in each sample was quantified by the standard plate counting assay to guarantee the bacteria amount is same in each group.

##### The qRT‐PCR Gene Detection Assay

At first, different nanoparticles (100 µL) were added into the *P. aeruginosa* biofilm cultured in 96‐well plates for 2 h, respectively. The biofilm suspension was taken out by pipette after a 10 min ultrasound, and then the bacteria in biofilm were obtained by centrifugation (3000 rpm, 5 min). Subsequently, the obtained bacteria were detected by the qRT‐PCR. Before detection, the bacteria amount in each sample was quantified by the standard plate counting assay to guarantee the bacteria amount is same in each group.

##### Statistical Analysis

Data are expressed as mean ± SD and the statistical significance is determined using one‐way ANOVA analysis. *P < 0.05, **P < 0.01.

## Conflict of Interest

The authors declare no conflict of interest.

## Supporting information

Supporting Information

## References

[1] a) C. D. Nadell , J. B. Xavier , K. R. Foster , FEMS Microbiol. Rev. 2009, 33, 206; 19067751 10.1111/j.1574-6976.2008.00150.x

[2] Y. Liu , H. C. van der Mei , B. R. Zhao , Y. Zhai , T. J. Cheng , Y. F. Li , Z. K. Zhang , H. J. Busscher , Y. J. Ren , L. Q. Shi , Adv. Funct. Mater. 2017, 27, 1701974.

[3] Y. Li , X. Liu , L. Tan , Z. Cui , D. Jing , X. Yang , Y. Liang , Z. Li , S. Zhu , Y. Zheng , K. W. K. Yeung , D. Zheng , X. Wang , S. Wu , Adv. Funct. Mater. 2019, 29, 1900946.

[4] a) H. F. Chambers , F. R. Deleo , Nat. Rev. Microbiol. 2009, 7, 629; 19680247 10.1038/nrmicro2200 PMC2871281

[5] S. M. Lehar , T. Pillow , M. Xu , L. Staben , K. K. Kajihara , R. Vandlen , L. DePalatis , H. Raab , W. L. Hazenbos , J. H. Morisaki , J. Kim , S. Park , M. Darwish , B. C. Lee , H. Hernandez , K. M. Loyet , P. Lupardus , R. Fong , D. Yan , C. Chalouni , E. Luis , Y. Khalfin , E. Plise , J. Cheong , J. P. Lyssikatos , M. Strandh , K. Koefoed , P. S. Andersen , J. A. Flygare , M. Wah Tan , E. J. Brown , S. Mariathasan , Nature 2015, 527, 323. 26536114 10.1038/nature16057

[6] a) B. Cao , F. Xiao , D. Xing , X. Hu , Small 2018, 14, 1802008; 10.1002/smll.201802008 30118562

[7] a) D. I. Andersson , D. Hughes , Nat. Rev. Microbiol. 2010, 8, 260. 20208551 10.1038/nrmicro2319

[8] S. Zhang , X.‐M. Xiao , F. Qi , P.‐C. Ma , W.‐W. Zhang , C.‐Z. Dai , D.‐F. Zhang , R.‐H. Liu , Chin. J. Polym. Sci. 2019, 37, 1105.

[9] a) L. Hall‐Stoodley , P. Stoodley , Cell. Microbiol. 2009, 11, 1034. 19374653 10.1111/j.1462-5822.2009.01323.x

[10] a) C. R. Arciola , D. Campoccia , P. Speziale , L. Montanaro , J. W. Costerton , Biomaterials 2012, 33, 5967. 22695065 10.1016/j.biomaterials.2012.05.031

[11] H. C. Flemming , J. Wingender , U. Szewzyk , P. Steinberg , S. A. Rice , S. Kjelleberg , Nat. Rev. Microbiol. 2016, 14, 563. 27510863 10.1038/nrmicro.2016.94

[12] a) R. I. Aminov , Environ. Microbiol. 2009, 11, 2970. 19601960 10.1111/j.1462-2920.2009.01972.x

[13] a) Y.‐W. Chang , A. A. Fragkopoulos , S. M. Marquez , H. D. Kim , T. E. Angelini , A. Fernández‐Nieves , New J. Phys. 2015, 17, 033017.

[14] B. Schaible , C. T. Taylor , K. Schaffer , Antimicrob. Agents Chemother. 2012, 56, 2114. 22290986 10.1128/AAC.05574-11 PMC3318321

[15] a) M. Yu , X. Xu , Y. Cai , L. Zou , X. Shuai , Biomaterials 2018, 175, 61. 29803104 10.1016/j.biomaterials.2018.05.019

[16] Y. Cheng , H. Cheng , C. Jiang , X. Qiu , K. Wang , W. Huan , A. Yuan , J. Wu , Y. Hu , Nat. Commun. 2015, 6, 8785. 26525216 10.1038/ncomms9785 PMC4659941

