# Supplementary material for: Relief of Biofilm Hypoxia Using an Oxygen Nanocarrier: A New Paradigm for Enhanced Antibiotic Therapy
Source: Adv Sci (Weinh). 2020 May 14;7(12):2000398. doi: 10.1002/advs.202000398 (PMC7312432; doi:10.1002/advs.202000398)
Supplement: Supplementary file 1 — Supporting Information [file ADVS-7-2000398-s001.pdf]

Copyright WILEY-VCH Verlag GmbH & Co. KGaA, 69469 Weinheim, Germany, 2020.

Supporting Information

for *Adv. Sci.,* DOI: 10.1002/advs.202000398

Relief of Biofilm Hypoxia Using an Oxygen Nanocarrier: A New Paradigm for Enhanced Antibiotic Therapy

Dengfeng Hu, Lingyun Zou, Weijiang Yu, Fan Jia, Haijie Han, Ke Yao, Qiao Jin, and Jian Ji**

Relief of Biofilm Hypoxia Using an Oxygen Nanocarrier: A New Paradigm for Enhanced Antibiotic Therapy

Dengfeng Hu[†], *Lingyun Zou*[†], *Weijiang Yu*[†], *Fan Jia*[†], *Haijie Han*[‡], *Ke Yao*[‡], *Qiao Jin*[†]* *and Jian Ji*[†]*

[†] MOE Key Laboratory of Macromolecule Synthesis and Functionalization of Ministry of Education, Department of Polymer Science and Engineering, Zhejiang University, Hangzhou, 310027, Zhejiang Province, PR China
[‡] Eye Center, Second Affiliated Hospital, School of Medicine, Zhejiang University, Hangzhou 310027, Zhejiang Province, PR China
* Corresponding authors: Qiao Jin (jinqiao@zju.edu.cn); Jian Ji (jijian@zju.edu.cn)

Experimental Section

Materials: Dioleoyl Phosphoethanolamine (DOPE), (2,3-Dioleoyloxy-propyl)-trimethylammonium (DOTAP), 1-palmitoyl-2-stearoyl-sn-glycero-3-phosphocholine (HSPC), 1,2-distearoyl-sn-glycero-3-phosphoethanolamine-N-[hydroxyl (polyethylene glycol)-2000] (DSPE-PEG2000) and cholesterol were obtained from A.V.T. Pharm. Ltd. (Shanghai, China). The 99% perfluorohexane (PFH) was purchased from Emergy Chemical Co., Ltd. (Shanghai, China). N-Acetyl-D-penicillamine, 3-(4,5-dimethylthiazol-2-yl)-2,5-diphenyl tetrazolium bromide (MTT) and 2-(4-amidino-phenyl)-6-indolecarbamidine dihydrochloride (DAPI) were provided by Sigma-Aldrich. All other reagents and solvents were of analytical grade and used as received without further purification.

Characterizations: The size and size distribution of nanoparticles are measured by dynamic light scattering (DLS) measurements by Zetasizer Nano-ZS from Malvern Instruments equipped with a He-Ne laser (633 nm) at 25 °C. The morphology of the nanoparticles is characterized by transmission electron microscopy (TEM) on a HT7700 TEM (Hitachi, Japan) at an accelerating voltage of 120 kV. Fluorescence measurements are made on a PTI QM-40 fluorescence spectrometer. Fluorescence images are observed by using a fluorescence microscope (Olympus IX81).

Measurement of PFH loading amount in lip@PFH@O_2: The PFH loading capacity in lip@PFH@O_2 is evaluated by gas chromatography-mass spectrometry (GC-MS) referring to the literature protocol.[1] lip@PFH nanoparticles (10, 20, 30, 40 μL) are dissolved in acetonitrile (40 μL) and vortex-mixed for 2 minutes. Then, 1,1,1,3,3-pentafluorobutane (200 μL) is added into each sample and the mixture is vortex-mixed for 5 minutes. The mixture is centrifuged (3000 rpm) for 2 min at 4 ℃ and then frozen at -30 ℃ for 2 hours. The lower phase is put into another 2 mL tube for GC-MS measurement and the upper phase is withdrawn.

Culturing and harvesting *P. aeruginosa* biofilm: *Pseudomonas aeruginosa* (abbreviated to *P. aeruginosa*, ATCC 27853, purchased from Guangdong culture collection center) is used in this research. At first, *P. aeruginosa* suspension (10^8 cfu/mL, 100 μL) and LB broth medium (100 μL) are placed into 24 well plates. The 24 well plates are then put into the nitrogen bag. Subsequently, the 24 well plates in nitrogen bag are put into the incubator to culture at 37 °C. After 24 hours, the timeworn medium is replaced by fresh LB broth medium and the biofilm

is grown in nitrogen condition for another 24 hours. Finally, the medium is removed and the *P. aeruginosa* biofilm attached on 24 well plates is obtained.

Penetration of lip@PFH into *P. aeruginosa* biofilm: Laser scanning confocal microscope (OLYMPUS IX83-FV3000) is employed to evaluate the penentration ability of nanoparticles into biofilms. Firstly, the lip@PFH is fluorescent marked by encapsulating the chlorin e6 (Ce6) inside. Next, the lip@PFH@Ce6 with red fluorescence under 561 nm exciting light is put into the *P. aeruginosa* biofilm for one hour. Afterwards, the biofilm is washed for three times with PBS solution to wash off the nanocarriers on the surface of biofilm and then the biofilm is stained by the SYTO 9, which can dye the live bacteria green, for 15 min in the dark.Then, the superfluous dye is wash off by PBS solution. At last, the biofilm is observed under laser scanning confocal microscope.

***In vivo* immunofluorescence staining of biofilm infected subcutaneous abscess by hypoxyprobe:** At first, different nanoparticles (100 μL) are added into the *P. aeruginosa* biofilms infected subcutaneous abscess for two hours respectively. Then, the pimonidazole hydrochloride (100 μL) is injected into the subcutaneous abscess for 90 minutes incubation. After incubation, the mice are sacrificed and the frozen abscess slices are stained through a reported procedure.[2] Finally, the slices are observed under a fluorescence microscope (Olympus IX81).

The *in vivo* qRT-PCR gene detection assay of bacteria in subcutaneous abscess: Firstly, different nanoparticles (100 μL) are injected into the *P. aeruginosa* biofilm infected subcutaneous abscess for two hours respectively. Then, the mice are sacrificed and the bacteria in abscess is obtained by centrifugation (3000 rpm, 5 min). Subsequently, the

obtained bacteria is detected by the qRT-PCR. Before detection, the bacterie amount in each sample is quantified by the standard plate counting assay to guarantee the bacteria amount is same in each group.

Statistical Analysis: Data were expressed as mean ± SD and the statistical significance is determined using one-way ANOVA analysis. *$P < 0.05$, **$P < 0.01$.

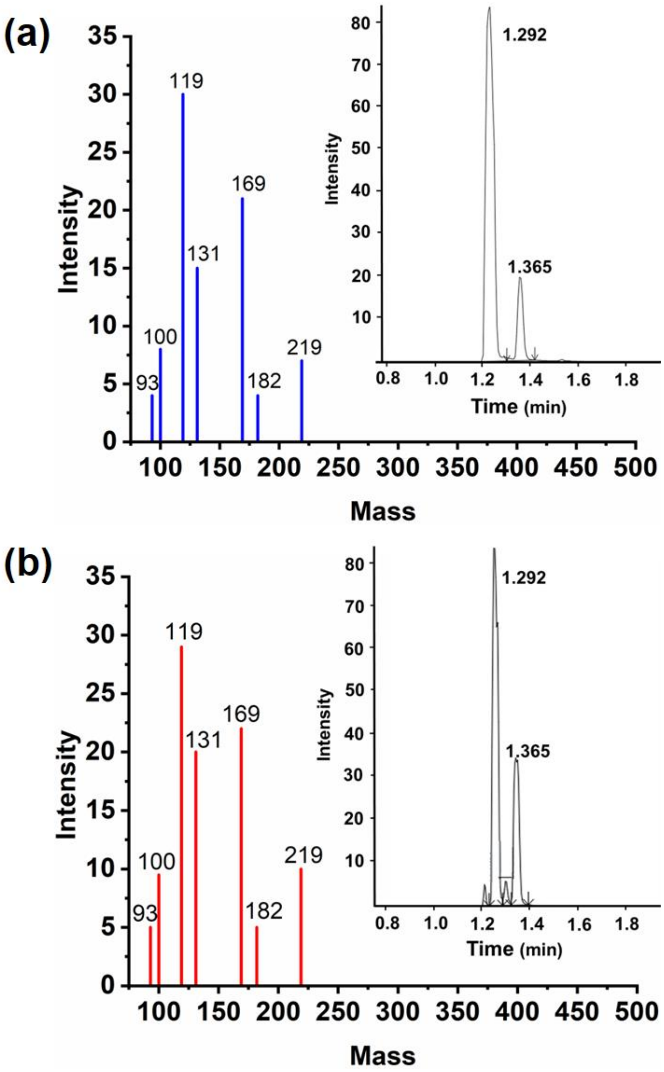

Figure S1. The mass spectrum peaks and efflux time of (a) pure PFH and (b) lip@PFH nanoparticles under the measurement of gas chromatography-mass spectrometry (GC-MS).

The samples are extracted by acetonitrile and 1,1,1,3,3-pentafluorobutane before GC-MS measurement referring to a literature protocol.[1]

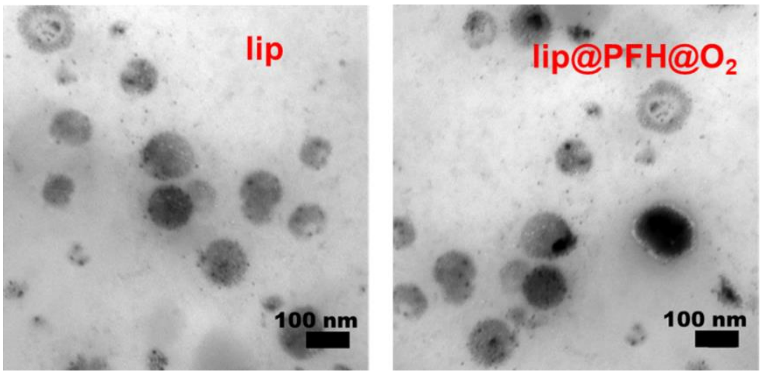

Figure S2. TEM images of lip and lip@PFH@O_2 nanoparticles.

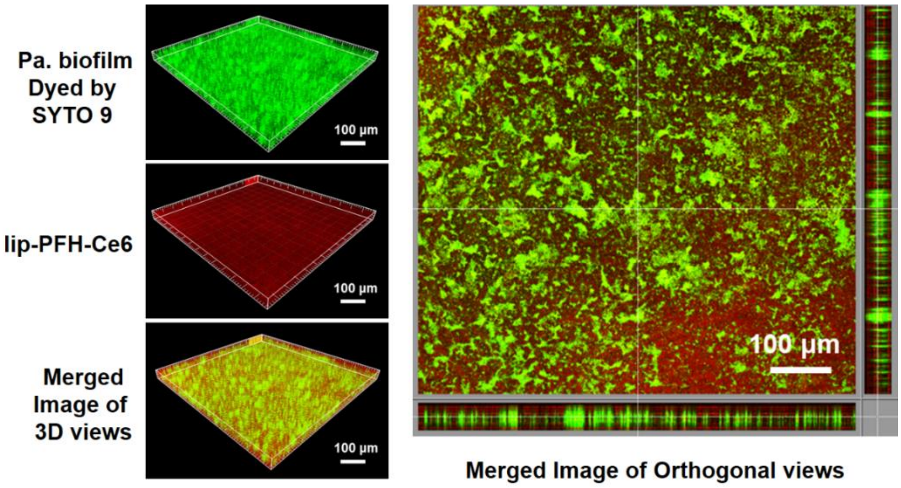

Figure S3. A) CLSM images of *P. aeruginosa* biofilm after incubation lip@PFH@Ce6 nanoparticles for one hour. (green fluorescence: *P. aeruginosa* biofilm stained by SYTO 9; red fluorescence: Ce6 in lip@PFH@Ce6). Scale bar: 100 μm.

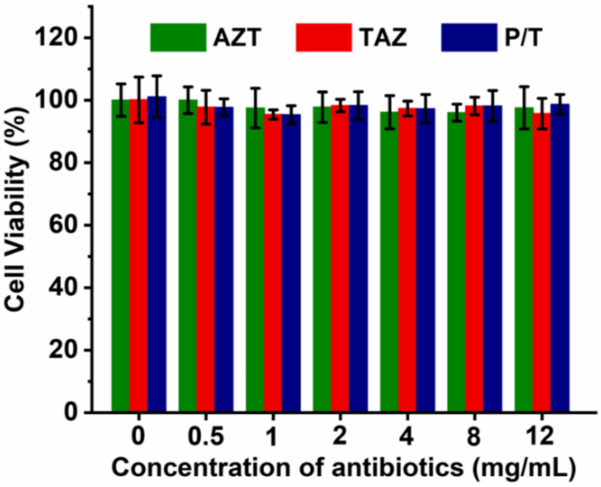

Figure S4. The *in vitro* cytotoxicity of NIH 3T3 fibroblast cells after incubation with AZT, TAZ and P/T with different concentrations.

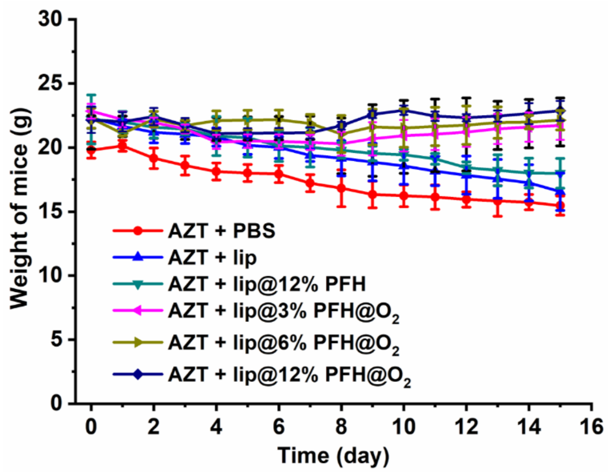

Figure S5. The weight change of mice with subcutaneous abscess after different treatments.

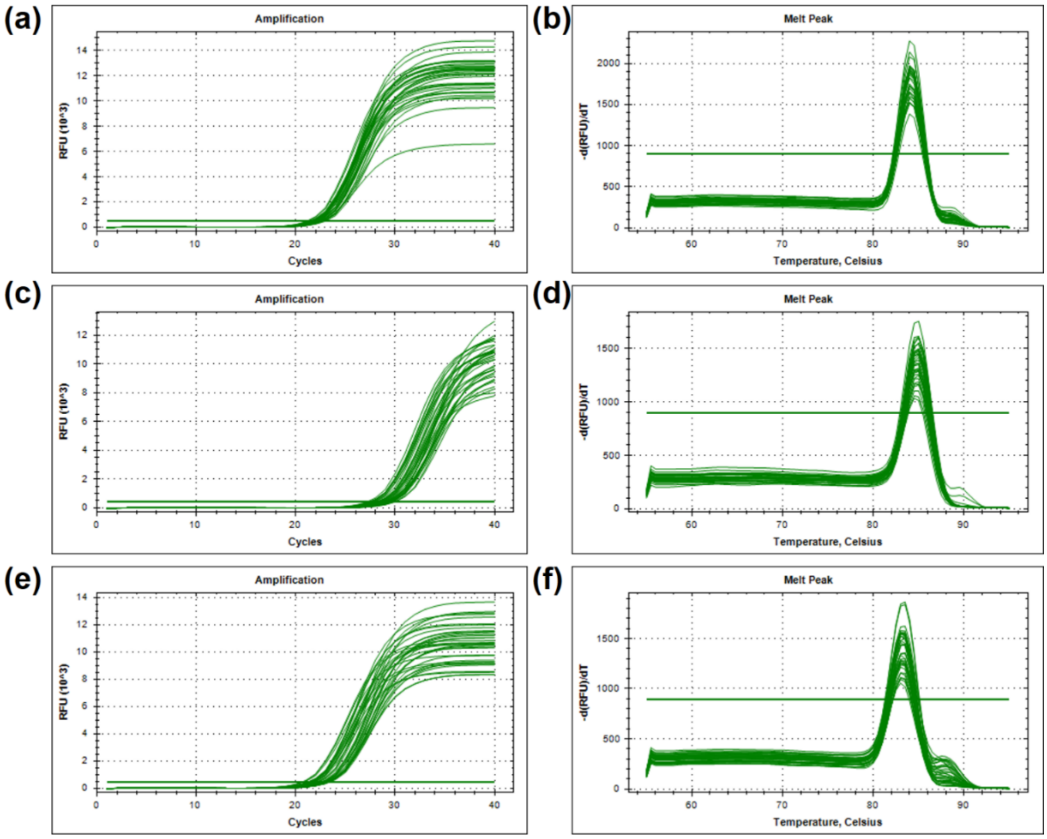

Figure S6. The amplification curve of (a) mexA, (c) mexC and (e) mexE in qRT-PCR gene detection; The melting-point curve of (a) mexA, (c) mexC and (e) mexE in qRT-PCR gene detection. The single peak indicates the gene is pure.

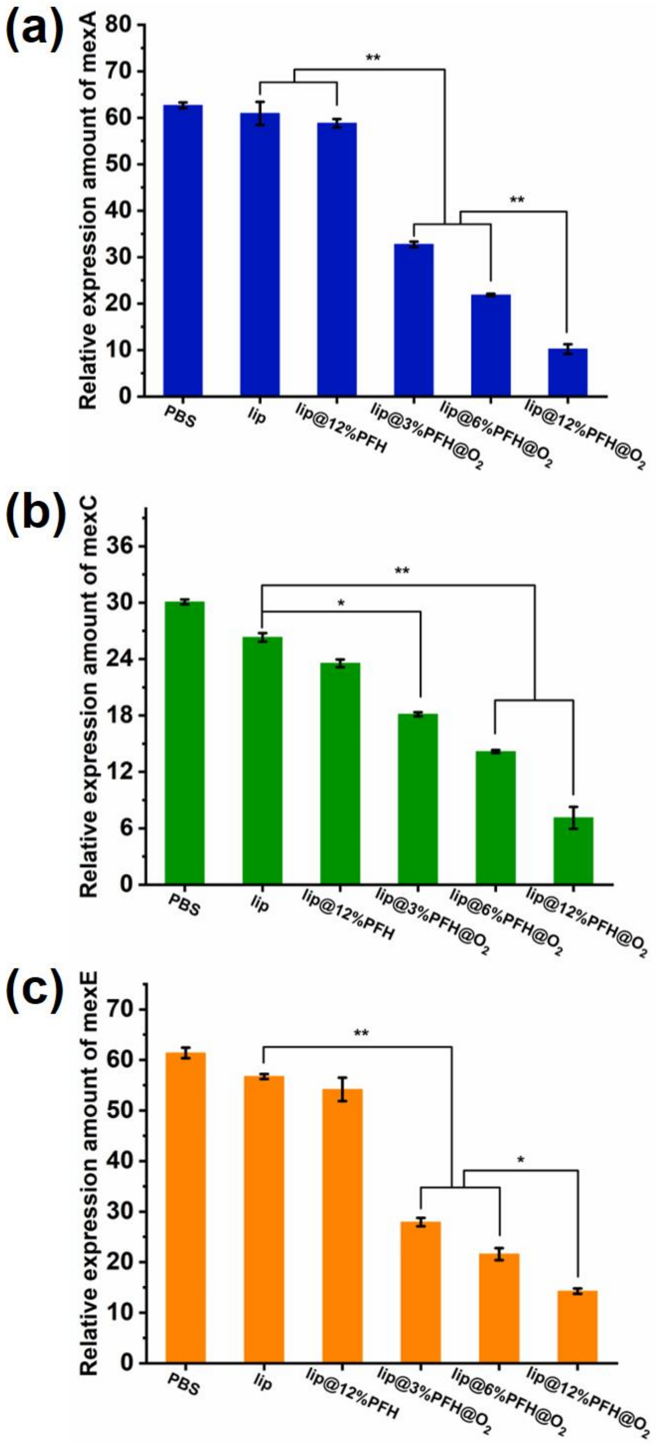

Figure S7. The qRT-PCR gene detection of relative expression amount of mexA, mexC and mexE in *P. aeruginosa* bacteria in biofilm treated with different groups in the subcutaneous *P. aeruginosa* biofilm infection model.

Reference

[1] a) X. Cai, X. Jia, W. Gao, K. Zhang, M. Ma, S. Wang, Y. Zheng, J. Shi, H. Chen, *Adv. Funct. Mater.* **2015**, *25*, 2520; b) G. Song, C. Liang, H. Gong, M. Li, X. Zheng, L. Cheng, K. Yang, X. Jiang, Z. Liu, *Adv. Mater.* **2015**, *27*, 6110.

[2] X. Zheng, X. Wang, H. Mao, W. Wu, B. Liu, X. Jiang, *Nat. Commun.* **2015**, *6*, 5834.
